# Palindromic Sequence Artifacts Generated during Next Generation Sequencing Library Preparation from Historic and Ancient DNA

**DOI:** 10.1371/journal.pone.0089676

**Published:** 2014-03-07

**Authors:** Bastiaan Star, Alexander J. Nederbragt, Marianne H. S. Hansen, Morten Skage, Gregor D. Gilfillan, Ian R. Bradbury, Christophe Pampoulie, Nils Chr Stenseth, Kjetill S. Jakobsen, Sissel Jentoft

**Affiliations:** 1 Centre for Ecological and Evolutionary Synthesis (CEES), Department of Biosciences, University of Oslo, Oslo, Norway; 2 Department of Medical Genetics, Oslo University Hospital, Oslo, Norway; 3 Fisheries and Oceans Canada, St. John's, Newfoundland, Canada; 4 Marine Research Institute, Reykjavík, Iceland; Natural History Museum of Denmark, University of Copenhagen, Denmark

## Abstract

Degradation-specific processes and variation in laboratory protocols can bias the DNA sequence composition from samples of ancient or historic origin. Here, we identify a novel artifact in sequences from historic samples of Atlantic cod (*Gadus morhua*), which forms interrupted palindromes consisting of reverse complementary sequence at the 5′ and 3′-ends of sequencing reads. The palindromic sequences themselves have specific properties – the bases at the 5′-end align well to the reference genome, whereas extensive misalignments exists among the bases at the terminal 3′-end. The terminal 3′ bases are artificial extensions likely caused by the occurrence of hairpin loops in single stranded DNA (ssDNA), which can be ligated and amplified in particular library creation protocols. We propose that such hairpin loops allow the inclusion of erroneous nucleotides, specifically at the 3′-end of DNA strands, with the 5′-end of the same strand providing the template. We also find these palindromes in previously published ancient DNA (aDNA) datasets, albeit at varying and substantially lower frequencies. This artifact can negatively affect the yield of endogenous DNA in these types of samples and introduces sequence bias.

## Introduction

Extensive degradation processes alter the base composition of historic and ancient DNA sequence data through biased sequence substitution and fragmentation. Specifically, enhanced cytosine deamination in single stranded 5′-overhangs leads to C-to-T substitutions at 5′-ends of sequences, and complementary G-to-A substitutions at 3′-ends [1]–[4], whereas fragmentation bias is evident through a relative increase of purines at the positions immediately preceding the 5′ termini [1], [5], [6]. Additionally, varying molecular approaches in next generation library creation protocols can further influence base composition of aDNA. For instance, the GC content is increased for fragments of shorter length [7]–[9], varying polymerases affect template length and GC content [10], bias against fragments starting with thymines residues has been observed using AT-overhang ligation protocols [11] and the typical complementary G-to-A substitutions at 3′-ends are absent when using protocols targeting single-stranded DNA (ssDNA) [6], [12], [13]. Such single-stranded protocols can also result in improved yield relative to background contaminant sequence [6], [12], [14], which is interesting because it indicates that the widely used protocols for double stranded DNA (dsDNA) suffer from an inherent inefficiency during library creation. While these comparisons provide valuable insights, there remains a gap in our understanding of the influence of library creation protocols on base composition bias and efficiency when handling ancient and historic samples.

Here, we identify an unusual abundance of misalignments at the 3′-end of Illumina reads derived from historic samples of Atlantic cod (*Gadus morhua*) that neither comply with known aDNA sequence damage patterns nor described methodological bias. Further characterization reveals that these misalignments consist of reverse complementary bases corresponding to the 5′-end of the same read, forming what we call an *interrupted palindrome*. Moreover, the inner sections of these palindromes align well to the reference genome and comprise naturally occurring sequence, whereas the terminal 3′-end bases align poorly. These specific characteristics strongly suggest that the interrupted palindromic sequences are generated by the formation of hairpin loops of single stranded DNA and that these hairpins are subsequently exposed to the activity of exonucleases, polymerases and ligases.

The typically short length distribution of historic and ancient DNA makes these fragments more susceptible to denaturation than modern DNA at lower temperatures [15]. We hypothesize that the potential for hairpin loops to form is sensitive to the differences in temperatures cycles that exist among alternative library creation protocols, because temperature affects both the denaturation of dsDNA into ssDNA and the subsequent stabilization of the hairpin loop stem. Importantly, successful amplification of ssDNA hairpin loops requires ligation of the appropriate sequencing adapters. Such opportunity is provided by forked adapters schemes – whereby the alternate adapters are located on opposing strands that are joined by a short complementary region – like those used by Illumina TruSeq protocols. We therefore characterize the presence of this phenomenon in two sets of libraries from historic samples that were created with protocols that differ in their temperature profile and use alternative adapter schemes. Additionally, we investigate the presence of these interrupted palindromes in previously published aDNA data. Based on our results, it appears that particular ligation protocols with forked adapter schemes are more susceptible to the formation and sequencing of this type of artifact in historic or ancient DNA. Such artifacts can negatively affect the yield of endogenous DNA, which can reduce the feasibility and economics of studies investigating historic and ancient DNA.

## Materials and Methods

### DNA extraction

DNA was extracted from scales and otoliths of Atlantic cod of different ages and localities (Table 1) following a protocol similar to [16]. Extractions were performed in a dedicated historic DNA facility at the Natural History Museum (NHM) in Oslo. In short, scales were incubated overnight and otoliths for three hours in TNES buffer (10 mM Tris, pH 7.5, 400 mM, NaCl 100 mM EDTA 0.6% SDS) with 5 mM CaCl_2_ and 10% proteinase K at 55°C. Extracts were concentrated using Amicon-30kDA Centrifugal Filter Units up to 100 µl after which DNA was purified with Qiaquick Nucleotide Removal Kit spin columns according to manufacturer's instructions. DNA was eluted in 50 µl of EB buffer at 37°C for 15 minutes and their quality was assessed on a Bioanalyzer 2100 instrument using the High sensitivity DNA kit (Agilent Technologies).

**Table 1:** Sample identity, location, year, library protocol and number of sequencing reads in millions (M) for historic Atlantic cod samples.

Sample ID	Location	Year	Library protocol	Untrimmed read pairs (M)	Trimmed and collapsed reads (%)	Average length (bp)	GC content (%)	Interrupted palindrome content (%)
4-83		1940		25.3	84	73	40	74
4-53	Iceland^a^	1940		18.1	87	67	37	70
6-50		1940		14.4	86	77	38	56
4-91		1940		21.9	87	77	38	70
W127		1949	TruSeq	16.2	87	67	35	71
W134		1949		26.6	87	74	38	78
1586		1959		16.5	86	79	37	57
676		1940		26.6	88	76	36	79
W131		1940		12.8	89	81	41	42
W135	Canada^b^	1949		13.7	88	75	41	59
W137		1949		11.2	88	63	41	57
690		1940		41.2	89	44	45	0.13
694		1940	Microplex	47.1	91	47	44	0.12
686		1940		41.2	95	56	44	0.17
704		1940		37.6	89	45	44	0.11

a Otolith samples.

b Scale samples.

Using AdapterRemoval [17], paired reads were trimmed for adapter sequence and collapsed based on sequence overlap. The average length in basepair (bp) and GC content (%) were calculated using the collapsed reads. Interrupted palindromes longer than three base pair were identified using the Python script clip_inverted_repeats.py.

### Library creation and sequencing

The TruSeq V2 DNA Sample Preparation kit (www.illumina.com) was followed using 100 to 250 ng of DNA from historical Atlantic cod samples according to the manufacturer's instructions with the following exceptions: First, no DNA fragmentation was performed. Second, all AMPure XP Beads clean-up steps before library amplification were replaced with MinElute PCR purification columns (Qiagen) to reduce the loss of smaller DNA fragments. Third, following the TruSeq ChIP Sample Preparation guide, a 5 min inactivation hold at 70°C was introduced after the 30 min 37°C A-Tailing incubation to reduce the presence of adapter dimer. Finally, adapter concentrations were 10-fold diluted. After adapter ligation, TruSeq libraries were amplified using the provided PCR primer and master mix as directed for 18 cycles.

The Microplex Library Preparation kit (www.diagenode.com) differs from the TruSeq protocol in that it is a single tube protocol, uses blunt-end ligation of stem-loop adapters, and no intermediate purification procedures are used before library amplification. None of the enzymes require temperatures higher than 55°C, and no high temperature holds are used to inactivate enzymatic activities. The Microplex kit was used according to the manufacturer's instructions using 40 to 50 ng of DNA, and entailed a total 14 cycles of PCR amplification.

After amplification, the quality and quantity of diluted library aliquots were assessed using the Bioanalyzer 2100 and Qubit dsDNA HS Assay (Life Technologies). Before multiplex sequencing, 11 individuals were pooled for the TruSeq libraries and four individuals were pooled for the Microplex libraries, respectively. The pooled TruSeq libraries were sequenced on two paired-end Illumina Hiseq 2000 lanes (100 bp) and the pooled Microplex libraries were sequenced on one paired-end lane according to manufacturer's instructions at the Norwegian Sequencing Centre (NSC: www.sequencing.no).

### Analysis

Using Illumina RTA & CASAVA software (versions 1.17.20 & 1.8.2, respectively) paired-end sequencing reads were demultiplexed and assigned to individual samples based on their index sequence, in which at most one mismatch was allowed. The vast majority of historic reads are smaller than 100 bp, thus forward and reverse reads can be joined based on sequence overlap. We used the program AdapterRemoval v1.5 [17] with –mm 0.33, –collapse, –trimns, –minlength 25 as settings to simultaneously collapse forward and reverse reads with a minimum overlap of 11 bases, and remove remaining adapter sequence. Quality scores of collapsed sequences were recalculated based on the best score of either the forward or reverse read, and only collapsed reads were used for further analyses. Collapsed reads were aligned to the Atlantic cod reference genome (ATLCOD1B, [18]) using the aln algorithm of BWA v.0.7.5a-r405 [19] with seeding disabled and -o 1 and -n 0.03, following recommendations in [20]. We only considered reads that align with a minimum mapping quality score (MapQ) of 25. Using SamTools v 0.1.19, SAM files were converted to BAM files and all files from the different samples were subsequently merged for the Illumina TruSeq and Microplex libraries, respectively. Patterns of sequence divergence from the reference genome were investigated using mapDamage v2.0.0 [4], [13]. We used the option –n to select a random subset of one million reads per merged dataset and the option –merge-reference-sequences to prevent excessive memory and disk usage. A limited number of reads mapping to the assembly were visually inspected using Tablet v 1.13.08.05 [21]. Using the Python script clip_inverted_repeats.py (File S2), we identified the presence of interrupted palindromes by determining the length distribution of sequence sections at the 3′-end that were a perfect reverse complement of the 5′-end of sequencing reads. In addition, reverse complementary sections of at least 4 bp were trimmed from the collapsed reads. We re-aligned the reads to the reference genome after this trimming procedure with BWA using identical settings as above and characterized the length distribution and GC content of reads *with* and *without* interrupted palindromes. Melting temperatures (Tm, at which one-half of the DNA is denatured) were calculated for Illumina TruSeq reads with and without these palindromes following the formula for sequences greater than 13 bp, unadjusted for salt concentration, as implemented in OligoCalc [22]. In addition, we assessed the clonality of these Illumina reads using the MarkDuplicates command from Picard Tools v. 1.96 (http://picard.sourceforge.net/).

Finally, using seqtk (https://github.com/lh3/seqtk version of Oct 16, 2012, commit hash d43d3704d4), we selected random subsets of one million untrimmed sequencing reads from publicly available aDNA studies [11], [23], [24] and from a paired-end library from the same specimen that was used for the Atlantic cod reference genome [18]. The contemporary Atlantic library was generated using the standard Illumina protocol that was size-fractionated to an average of 180 bp insert lengths. These subsets of reads were treated similarly with regard to adapter trimming and treatment of paired-end data, alignments towards their respective reference genomes, and the detection and the trimming of reverse complement sections. The aDNA data studies were selected based on the use of comparable library preparation protocols targeting double stranded DNA, the access to original, untrimmed raw sequences and the availability of a reference genome.

## Results

**Figure 1:**
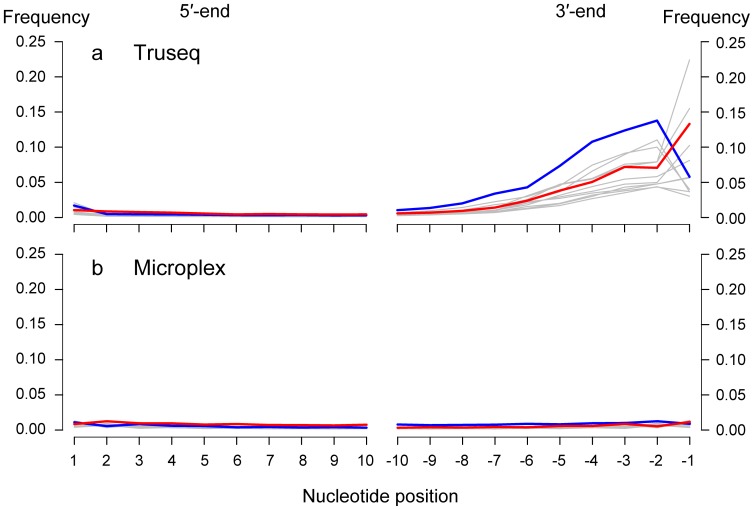
Frequency of nucleotide substitutions along historic reads of Atlantic cod. Reads were generated using the TruSeq V2 library creation protocol (**a**) or the Microplex single tube protocol (see methods) (**b**). Misalignments to the reference at the 5′ and 3′-end of sequencing reads are the result of elevated proportions of C to T substitutions (red), G to A substitutions (blue) and other possible substitutions (grey). The figure was generated using the program mapDamage V2.0.0 using 1 million randomly chosen reads for merged Illumina and Microplex libraries [13].

The three Illumina HiSeq 2000 lanes generated a minimum of 11 million read pairs per sample (Table 1). On average, sequenced reads from TruSeq libraries were longer than those from Microplex libraries, likely due to an absence of intermediate cleaning steps in the Microplex protocol, promoting a greater retention and amplification of smaller fragments. The TruSeq libraries had a lower overall GC content compared to the Microplex libraries and compared to the GC content of the Atlantic cod genome assembly (45%, [18]). Strikingly, the TruSeq libraries show elevated levels of sequence divergence with the reference genome through a unidirectional increase of all nucleotide substitution rates at their 3′-end (Figure 1). These increased substitution rates do not appear at the TruSeq 5′-end nor at the sequence ends of the Microplex libraries. For the TruSeq 5′-end and the Microplex libraries, levels of sequence divergence – in particular the typical C-to-T at 5′-ends and G-to-A substitutions at 3′-ends – were lower than 3% (Figure 1, see also Figure S1 in File S1). These levels are comparable to samples of similar age (e.g. see [24]) for which polymerases were used that are not able to replicate through uracil.

**Figure 2:**
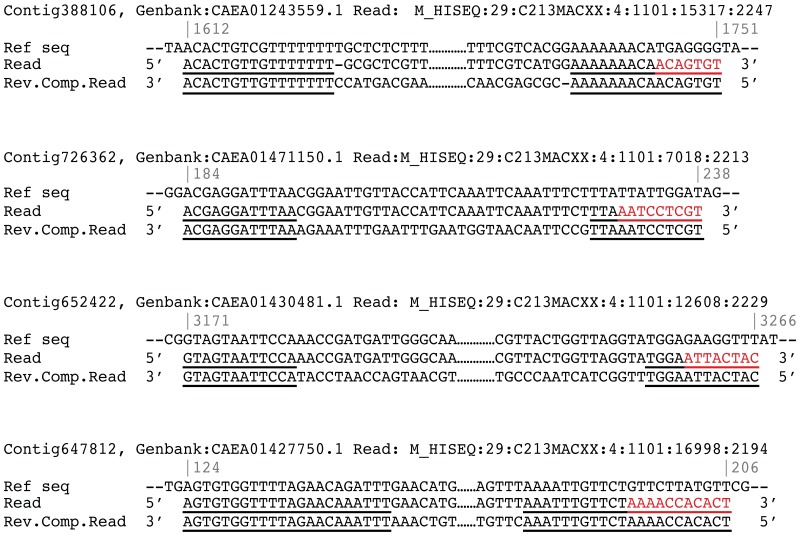
Alignments of reads containing interrupted palindromes to the Atlantic cod reference genome. The entire reverse complement sequence (Rev.Comp.Read) is displayed underneath the original read. Interrupted palindromes occur at read ends (underlined), and extensive misalignments (red) to the reference occur most proximate to the 3′-end of the read. For display purposes, alignments that did not fit a single line are clipped in the middle of the sequence (indicated with dots, not to scale). The relative start and end position of the alignments are shown above the reference sequence (grey numbers).

**Figure 3:**
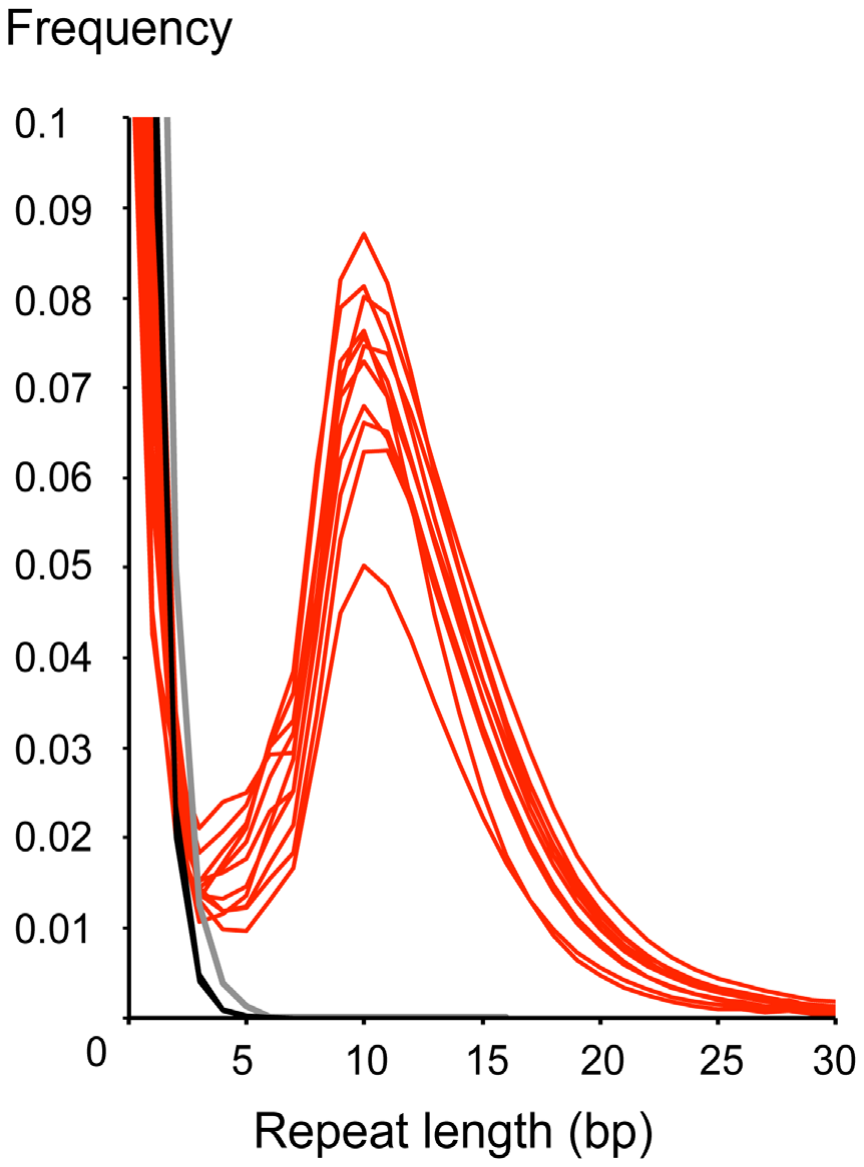
Length distribution of interrupted palindromes at 5′ and 3′-ends in Illumina HiSeq 2000 reads of Atlantic cod (*Gadus morhua*). Reads were generated from 11 historic samples using TruSeq library creation protocols (red lines), four historic samples using Microplex protocols (black lines) and one modern sample using TruSeq protocols (grey line). Terminal palindromic sequences longer than three basepair are rare in the Microplex and modern samples.

Visual inspection of the sequence data showed that a proportion of reads from the TruSeq libraries had terminal reverse complement bases at the 3′-end, which form an interrupted palindrome with bases at the 5′-end. These terminal palindromic sequences align well at the 5′, yet only partially at the 3′-end when compared to the reference genome (See Figure 2 for typical examples). More specifically, the 3′-end palindrome consists of two sections of which only the section most proximate to the 3′-end does not align properly. The length of terminal palindromes detectable in Microplex or TruSeq libraries generated from contemporary DNA was generally less than or equal to 3 bp. However, in TruSeq libraries generated from historical DNA, there was a second group of palindromes, with an average length of 11 bp (Figure 3), which occur in 42 to 78 percent of the collapsed reads (Table 1). These reads with interrupted palindromes have different properties compared to those without: First, their total average length is shorter than those containing no palindromes (Figure S2 in File S1). Second, reads with palindromes longer than three bp have a lower GC content, regardless of read length (Figure S3 in File S1). Third, Illumina reads with palindromes have a lower average melting temperature (62°C) than those without (74°C, Figure S4 in File S1). Finally, approximately 3 to 37 fold the number of duplicate reads is observed in reads with palindromes compared to those without (Table S1 in File S1), indicative of an increased tendency for clonal amplification. It is possible that the relatively short length of the palindromic reads promotes preferential amplification leading to such increased levels of clonality (e.g. see [10]).

**Figure 4:**
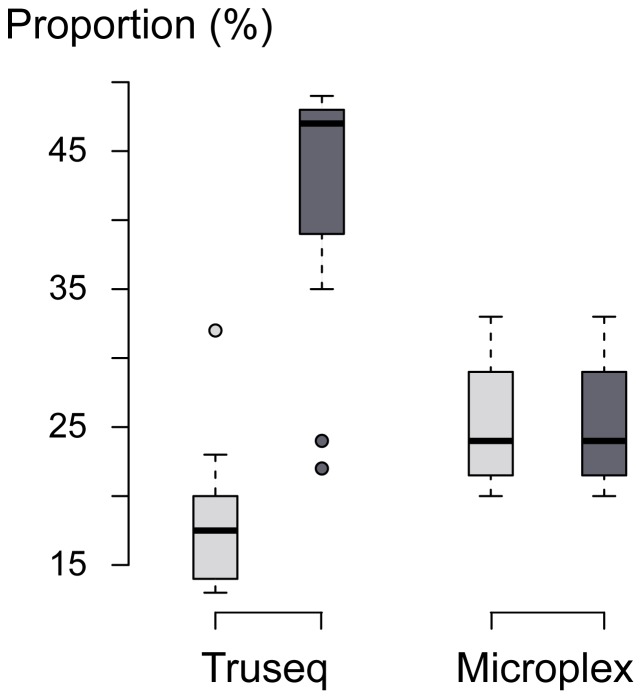
Proportion of reads aligning to the Atlantic cod genome for TruSeq and Microplex libraries. The proportions of reads aligning (relative to the number of untrimmed read pairs) were calculated for libraries including interrupted palindromes (light grey) and those for which these palindromes (dark gray) were removed at the 3′-end. Only reads with a minimum mapping quality (MapQ) value of 25 were considered.

The BWA aln algorithm only allows alignments with a maximum proportion of nucleotide mismatches between read and reference, determined by the option –n. Because the number of mismatches is increased by the divergent sections of the 3′-end palindromes, it can be expected that their presence lowers the proportion of reads that aligns to the reference genome. Indeed, by removing the terminal palindromic bases from the 3′-end of reads, more than double the number of reads aligned with a minimum MapQ value of 25 for the TruSeq libraries, while no such difference was observed for the Microplex libraries (Figure 4). These results strongly indicate that the nucleotide divergence at the 3′-end is artificial, and that it is a common artifact associated with Atlantic cod endogenous DNA. Interestingly, after removing the terminal palindromes, proportionally more reads could be aligned using the TruSeq libraries compared to the Microplex libraries. Two characteristics of the Microplex libraries may explain this reduced number of high quality alignments. First, Microplex libraries contain a higher abundance of shorter reads (Table 1). Moreover, Microplex reads contain substantially more dinucleotide repeats than the contemporary or historic TruSeq libraries (Figure S5 in File S1). Both these characteristics likely reduce the number of high quality alignments for this type of library.

**Figure 5:**
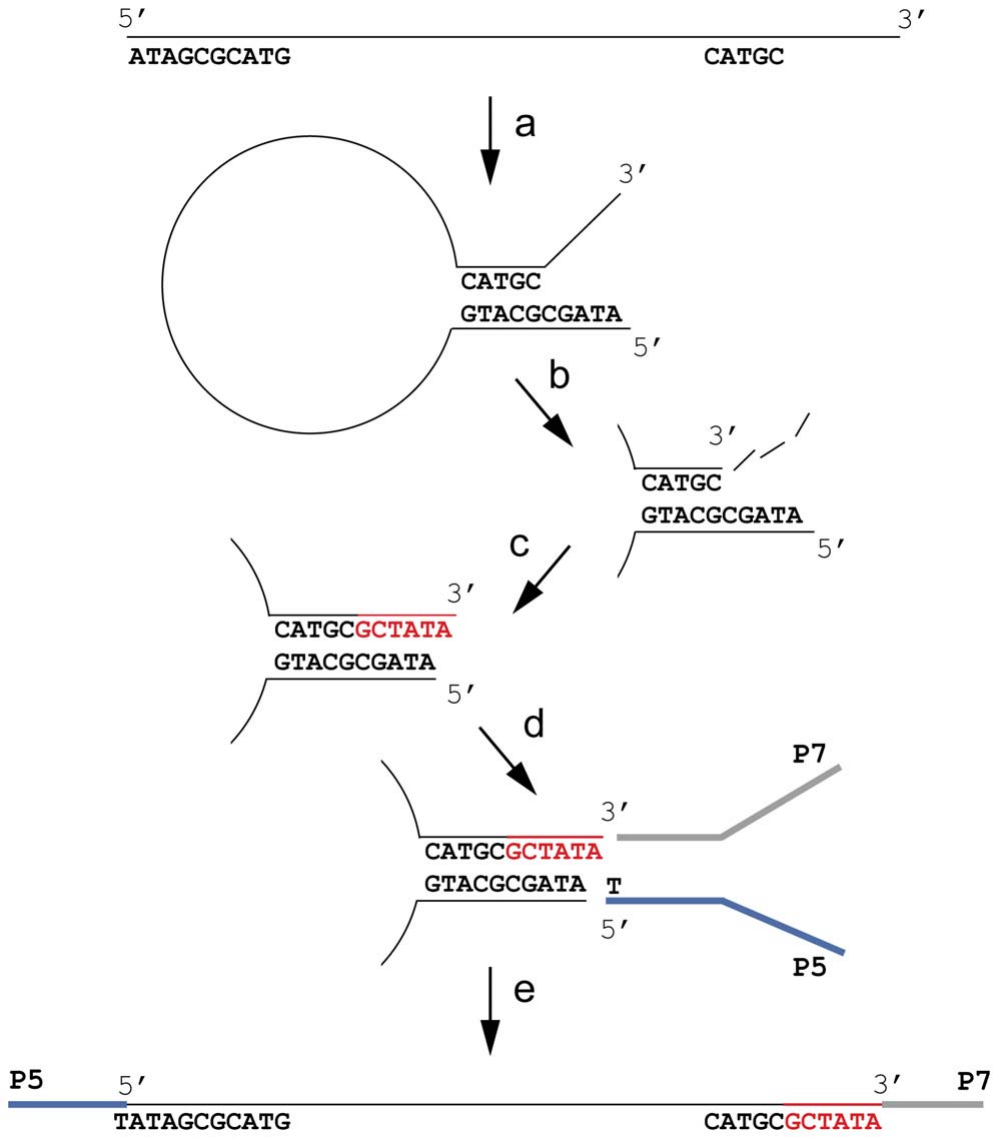
Hypothetical process creating interrupted palindromes during library creation for next generation sequencing. Single stranded DNA forms a hairpin loop through the presence of short, naturally occurring reverse complement sequences (**a**). Exonuclease activity removes unannealed 3′-ends if present, creating a 5′ overhang (**b**). Polymerases extend the 3′ strand based on the 5′-end of the same strand and create an A-overhang (red, **c**). Forked Illumina adapters, P5 (blue) and P7 (grey) are ligated to the double stranded stem, using AT-overhang ligation (**d**). The denatured, ligated construct is suitable for amplification by PCR, and the artificially extended sequence results in a reverse complement artifact that covers both ends of the strand (**e**).

The scarcity of interrupted palindromes longer than three basepair in the Microplex reads compared to their abundance in the TruSeq libraries strongly suggests that these artifacts are methodological. Moreover, the typical, unidirectional occurrences of artificial sequence at the 3′-end indicate a likely process by which these artifacts are generated. We propose that single-stranded DNA forms a hairpin loop based on short, naturally occurring reverse complement sequences (Figure 5). This hairpin loop is subsequently exposed to the activity of 3′ to 5′ exonucleases, removing unannealed 3′-ends if present. Polymerases then fill in 3′ recessed ends (5′ overhang) by catalyzing DNA in the 5′ to 3′ direction. Importantly, such directional activity always extends the hairpin loop using the 5′ strand as template, but not the 3′ strand. After blunt-end repair and A-overhang extension of the hairpin loop stem, ligation of the forked P5 and P7 Illumina adapters allows the replication of the construct through PCR.

The presence of interrupted palindromic sequence in Illumina libraries was investigated using data from three previously published aDNA studies [11], [23], [24] using our clip_inverted_repeats.py program (Table 2). The relative abundance of these palindromes varies among and within studies, and is overall lower than those reported in our current study. For the Rasmussen et al. 2010 study, reads with library IDs that contain SAQ and HUMefd have consistently higher proportions of palindromes than those libraries containing HUMgjn though the same library protocols were used. For the Sequin-Orlando et al. 2013 study, identical samples generated a higher abundance of palindromes using an A-overhang (AT) protocol with forked adapters compared to using a blunt-end (BE) protocol without such adapters. We observe a highly linear relationship (R^2^ = 99%, Figure S6 in File S1) between the number of reads with palindromes and the increase in number reads that align (with MapQ values greater than 25) to their respective reference genomes after 3′-end palindrome trimming, indicating that this phenomenon affects endogenous DNA in these samples.

**Table 2:** The abundance of interrupted palindromes in sequencing reads from ancient and historic specimens.

Study and sample type	SRA ID	Library ID	Palindromes (%)
	SRR031056	SAQ	10.5
	SRR031061	SAQ	7.2
	SRR031063	SAQ	8.1
	SRR030833	HUMefdRAH	7.9
	SRR030853	HUMefdRAH	7.5
	SRR030873	HUMefdRAH	6.4
Whole genome sequencing	SRR030875	HUMefdRAH	7.0
(WGS) of an ∼4000 year old	SRR030867	HUMefdRAH	7.6
paleoeskimo from Greenland^1^ +	SRR030923	HUMefdRCN	5.1
	SRR030942	HUMefdRCO	5.7
	SRR030949	HUMgjnRALDAAAPEI	0.4
	SRR031000	HUMgjnRALDAAAPEI	0.4
	SRR031001	HUMgjnRALDAAAPEI	0.4
	SRR031044	HUMgjnRAFDAAAPEI	0.3
	SRR030971	HUMgjnRACPEI	0.6
	SRR031029	HUMgjnRAXPEI	0.4
	SRR188204	HUMixgRAGSEIW	0.3
WGS of a 100 year old	SRR188192	HUMixgRAFSEW	0.5
Aboriginal Australian^2^ +	SRR188177	HUMgspRBFSEW	0.3
	SRR188174	HUMgspRBASEW	0.4
Ligation bias study, amongst	SRR959263	E.q Quagga *AT*	1.3
others based on WGS of a	SRR959261	E.q Quagga *BE*	0.3
Quagga museum specimen and	SRR959266	H. Saldiasi *AT*	2.5
an ∼11500 year old Hippidion^3^	SRR959264	H. Saldiasi *BE*	0.3

1 Rasmussen et al. 2010 using the TruSeq Sample Preparation kit (Illumina.com).

2 Rasmussen et al. 2011 using the Rapid Library kit from Roche-454 (Branford, CO) and Illumina adapter mix.

3 Sequin-Orland et al. 2013 using the NEBNext Quick DNA Library Prep Master Mix Set for 454 (New England BioLabs, ref E6090 for A-tailed (*AT*) ligation of forked Illumina adapters and the NEBNext DNA Library Prep Master Mix Set for 454 (New England BioLabs, ref E6070) for blunt-end (*BE*) ligation of Illumina adapters without forked shape.

+ A subset of available aDNA libraries was inspected for artifacts.

Only palindromic sequences longer than three basepair and alignments with a minimum MapQ of 25 are reported.

## Discussion

Here we report the occurrence of interrupted palindromes at the 5′ and 3′-ends of sequencing reads from historic and ancient DNA. Several lines of evidence suggest that hairpin loop formation of ssDNA underlies these palindromes. Most importantly, reads with palindromes show extensive misalignments, yet solely at their 3′-ends. This pattern specifically fits the directional activities of commonly used 3′ to 5′ exonucleases, followed by the syntheses of DNA in the 5′ to 3′ direction by polymerases (e.g. see [1]). Such directional enzymatic activities result in the exclusive extension of erroneous, reverse complement nucleotides at the 3′-end, using the 5′ strand as template. In addition, the inner sections of the interrupted palindromes align well to the reference genome and consist of endogenous DNA. The high abundance of reads containing terminal palindromes highlights the natural potential of these endogenous short sections of palindromic sequence to form hairpin structures given appropriate conditions. Finally, the higher AT-content and lower melting temperatures of the reads containing interrupted palindromes reflect their increased predisposition to form ssDNA for a given temperature. Taken together, these observations support the hypothesis that hairpin loop formation of ssDNA allows the proliferation of 3′-end terminal palindromes during the library creation of ancient and historic DNA.

The final abundance of interrupted palindromes in ancient and historic sequencing libraries is likely the result from a complex combination of factors. Most importantly, forked adapter schemes – such as those used by the Illumina TruSeq V2 protocol or similar – provide the necessary adapters to ssDNA hairpin loops allowing their successful amplification. Apart from this characteristic, variation in enzyme properties, temperature profiles, amplification conditions, template length and GC content may all influence the efficiency of hairpin loop proliferation. The TruSeq V2 protocol for instance, creates the A-overhang at 37°C, whereas others (e.g. the NEBNext Quick DNA Library Prep Master Mix for 454, New England BioLabs, ref E6090 or the Rapid Library Preparation kit, Roche, Ref 05608228001) use a Taq polymerase at 72°C. Therefore, in the Illumina TruSeq protocol all enzymatic steps from blunt-end repair to ligation are performed at relatively low temperatures. It is possible that the lower temperatures used by the TruSeq protocol promote the formation of hairpin loops in ssDNA, allowing these constructs to be exposed to the activity of exonucleases, polymerases and ligases. For instance, we find substantially lower palindrome formation in libraries whereby A-overhangs were created at 72°C, despite the use of forked adapters [11], [24]. Moreover, there is extensive variation in palindrome abundance among identically generated libraries of the same sample [23], indicating that subtle changes in protocol contribute to this phenomenon. Another feature of reads with interrupted palindromes is a relative increase in clonality. This increase indicates that these reads can be preferentially amplified once adapters have been ligated to the hairpin loop. Interestingly, forked adapters are usually ligated using A-overhang protocols. This type of ligation protocol has also been found to bias against DNA templates starting with thymine residues [11]. Thus, considering the potential complexities and bias associated with A-overhang ligation and this specific adapter scheme, it appears that library creation protocols that use blunt-end ligation without forked adapters, or those that specifically target single-strand DNA are more suited for degraded DNA of historic and ancient origin.

We highlight several factors that may have specifically promoted the high abundance of interrupted palindromes in our data. It is possible that our samples contained a high abundance of ssDNA before the initiation of library creation. For example, our samples were incubated with Proteinase K at a temperature of 55°C, which can have contributed to the denaturation of dsDNA. Nevertheless, the majority of reads with interrupted palindromes have a melting temperature above this value; hence we presume it unlikely that this step is the sole cause for the proliferation of ssDNA. Alternatively, it is possible that our library protocol has generated additional ssDNA reads. One alteration to our protocol compared to the TruSeq protocol of Rasmussen et al. 2010 is the introduction of a 70°C hold that was introduced after A-tailing to reduce the presence of adapter dimer. The high temperature hold occurs right before ligation of the adapters and may introduce additional ssDNA at this stage. Following this scenario, ssDNA would be generated *after* the cleanup of T4 DNA polymerases during the inactivation of A-tailing polymerases. Nevertheless, most commercially available ligases possess detectable exonuclease activity [25], which are introduced after the A-tailing inactivation step. It is possible that the temperature hold used here was sufficient for denaturing dsDNA, but not sufficient to inactivate all A-tailing polymerases. The combination of contaminating exonuclease activity and remaining activity of polymerases could then further contribute to the creation of hairpin loops and subsequent palindrome formation.

The extensive presence of reads with interrupted palindromes introduces sequence bias and can negatively affect yield when processing historic and ancient DNA. Sequence bias is introduced because palindromic reads are AT-rich, whereas yield is reduced if measures must be introduced to remove the artifacts themselves. Moreover, it can be challenging to identify and remove such artifacts. Our clip_inverted_repeats.py program currently relies on the detection of perfect reverse complement sections, and hence fails to detect their presence if sequence errors occur at read ends, or if adapter sequence is incompletely trimmed. The number of artifacts detected here may therefore represent an underestimation. Since reads that contain artifacts are more difficult to align to a reference genome using a program like BWA aln, the proportion of endogenous DNA is potentially underestimated. Finally, we found evidence for the presence of ssDNA by observing reverse complement sequence artifacts in our current study. Yet an absence of interrupted palindromes does not necessarily reflect an absence of ssDNA; the use of high temperatures steps during library creation before adapter ligation can nonetheless result in the denaturation of dsDNA. Such denaturation process not only biases template composition, but may also reduce sequence yield if limited amounts of endogenous template is available. Our finding of interrupted palindromes highlights the sensitivity of highly fragmented, short ancient and historic dsDNA towards temperature driven denaturation.

## Supporting Information

File S1 **Supporting Tables, Supporting Figures and associated references.**
(DOC)

File S2 **Python script** “**clip_inverted_repeats.py”, which identifies reverse complementary basepairs at the beginning and end of next generation sequencing reads.**
(PY) Click here for additional data file.
