# Correction: Palindromic Sequence Artifacts Generated during Next Generation Sequencing Library Preparation from Historic and Ancient DNA

**DOI:** 10.1371/journal.pone.0103170

**Published:** 2014-07-29

**Authors:** The PLOS ONE Staff

There are errors in Tables 1 and 2. Several column border lines were removed during the production process. Please find corrected versions of each table below. The publisher apologizes for this error.

**Table 1:** Sample identity, location, year, library protocol and number of sequencing reads in millions (M) for historic Atlantic cod samples. Using AdapterRemoval [17], paired reads were trimmed for adapter sequence and collapsed based on sequence overlap. The average length in basepair (bp) and GC content (%) were calculated using the collapsed reads. Interrupted palindromes longer than three base pair were identified using the Python script clip_inverted_repeats.py.

Sample ID	Location	Year	Library protocol	Untrimmed read pairs (M)	Trimmed and collapsed reads (%)	Average length (bp)	GC content (%)	Interrupted palindrome content (%)
4-83		1940		25.3	84	73	40	74
4-53	Iceland^a^	1940		18.1	87	67	37	70
6-50		1940		14.4	86	77	38	56
4-91		1940		21.9	87	77	38	70
W127		1949	TruSeq	16.2	87	67	35	71
W134		1949		26.6	87	74	38	78
1586		1959		16.5	86	79	37	57
676		1940		26.6	88	76	36	79
W131		1940		12.8	89	81	41	42
W135	Canada^b^	1949		13.7	88	75	41	59
W137		1949		11.2	88	63	41	57
690		1940		41.2	89	44	45	0.13
694		1940	Microplex	47.1	91	47	44	0.12
686		1940		41.2	95	56	44	0.17
704		1940		37.6	89	45	44	0.11

a Otolith samples.

b Scale samples.

**Table 2:** The abundance of interrupted palindromes in sequencing reads from ancient and historic specimens. Only palindromic sequences longer than three basepair and alignments with a minimum MapQ of 25 are reported.

Study and sample type	SRA ID	Library ID	Palindromes (%)
Whole genome sequencing (WGS) of an ∼4000 year old paleoeskimo from Greenland^1+^	SRR031056	SAQ	10.5
	SRR031061	SAQ	7.2
	SRR031063	SAQ	8.1
	SRR030833	HUMefdRAH	7.9
	SRR030853	HUMefdRAH	7.5
	SRR030873	HUMefdRAH	6.4
	SRR030875	HUMefdRAH	7.0
	SRR030867	HUMefdRAH	7.6
	SRR030923	HUMefdRCN	5.1
	SRR030942	HUMefdRCO	5.7
	SRR030949	HUMgjnRALDAAAPEI	0.4
	SRR031000	HUMgjnRALDAAAPEI	0.4
	SRR031001	HUMgjnRALDAAAPEI	0.4
	SRR031044	HUMgjnRAFDAAAPEI	0.3
	SRR030971	HUMgjnRACPEI	0.6
	SRR031029	HUMgjnRAXPEI	0.4
WGS of a 100 year old Aboriginal Australian^2+^	SRR188204	HUMixgRAGSEIW	0.3
	SRR188192	HUMixgRAFSEW	0.5
	SRR188177	HUMgspRBFSEW	0.3
	SRR188174	HUMgspRBASEW	0.4
Ligation bias study, amongst others based on WGS of a Quagga museum specimen and an ∼11500 year old Hippidion^3^	SRR959263	E.q Quagga *AT*	1.3
	SRR959261	E.q Quagga *BE*	0.3
	SRR959266	H. Saldiasi *AT*	2.5
	SRR959264	H. Saldiasi *BE*	0.3

1 Rasmussen et al. 2010 using the TruSeq Sample Preparation kit (Illumina.com).

2 Rasmussen et al. 2011 using the Rapid Library kit from Roche-454 (Branford, CO) and Illumina adapter mix.

3 Sequin-Orland et al. 2013 using the NEBNext Quick DNA Library Prep Master Mix Set for 454 (New England BioLabs, ref E6090 for A-tailed (*AT)* ligation of forked Illumina adapters and the NEBNext DNA Library Prep Master Mix Set for 454 (New England BioLabs, ref E6070) for blunt-end (*BE*) ligation of Illumina adapters without forked shape.

+ A subset of available aDNA libraries was inspected for artifacts.
