# Supplementary material for: Palindromic Sequence Artifacts Generated during Next Generation Sequencing Library Preparation from Historic and Ancient DNA
Source: PLoS One. 2014 Mar 7;9(3):e89676. doi: 10.1371/journal.pone.0089676 (PMC3946424; doi:10.1371/journal.pone.0089676)
Supplement: File S1 — Supporting Tables, Supporting Figures and associated references. (DOC) [file pone.0089676.s001.doc]

**Figure S1.** Frequency of nucleotide substitutions along historic reads of Atlantic cod.

Reads were generated using the TruSeq V2 library creation protocol and terminal interrupted palindromes were removed before mapping to the reference genome with BWA v.0.7.5a-r405 . Misalignments to the reference at the 5′ and 3′-end of sequencing reads are the result of elevated proportions of C to T substitutions (red), G to A substitutions (blue) and other possible substitutions (grey). The figure was generated using the program mapDamage V2.0.0 using 1 million randomly chosen reads (using the option –n).

**Figure S2.** Length distribution of Illumina TruSeq reads from historic Atlantic cod samples.

The average read length of sequences with an interrupted palindrome longer than 3 bp (red) is shorter than those of sequences without a palindrome (black).

**Figure S3.** Average GC content for Microplex and libraries from historic Atlantic cod samples calculated per read length.

Reads were divided into those *with* and *without* interrupted palindromes greater than 3bp. Read length and GC content were calculated after the 3′-end terminal palindrome was removed. For the Microplex libraries with interrupted palindromes all detected reads were used (*n* = 200755), otherwise a subset of 500 thousand reads was randomly selected to calculate the average.

**Figure S4.** Frequency distribution of melting temperatures for Illumina TruSeq reads from historic Atlantic cod samples

Reads were divided into those *with* (red) and *without* (black) interrupted palindromes greater than 3bp. Basic melting temperatures -unadjusted for salt concentration- for sequences longer than 13bp were calculated following the formula Tm= 64.9 +41*(yG+zC-16.4)/(wA+xT+yG+zC) as implemented in OligoCalc, where *w,x,y,z* are the number of the bases A,T,G,C in the sequence, respectively . 3’-end terminal palindromes greater than 3bp were removed prior to calculating melting temperatures.

**Figure S5.** The proportion of dinucleotide repeats in Atlantic cod sequencing reads.

Contemporary (grey) and historic (dark grey) TruSeq libraries contain less AC and AG dinucleotide repeats (and their complementary base pairs CT and GT, respectively) than Microplex libraries. All libraries were clipped for 3′-end terminal palindromes. FastQ files were converted to FastA files using seqtk (<https://github.com/lh3/seqtk> version of Oct 16, 2012, commit hash d43d3704d4) after which dinucleotide repeat content for all reads was determined using the program Tandem Repeat Finder (TRF, version 407b, ), with the following parameters: - *2 2 7 80 10 10 2* and the option –ngs. The first 3 parameters (*2, 2, 7*) provide standard weights for match, mismatch and indel penalties following Smith-Waterman style local alignment. Parameter four and five (*80, 10*) provide standard probability values. Parameters six and seven (*10, 2*) determine minimum alignment score and max period, respectively. Using these settings, dinucleotide repeats with a minimum length of 5 basepair (e.g. ACACA) were identified. The option –ngs eases downstream data manipulation through reduced data output. The number of identical dinucleotide repeats that initiate with alternative bases (e.g. either ACACA or ACACA) were summed and their percentage was calculated by dividing this number by the total number of bases in the FastQ file multiplied by 100. Stretches of single nucleotide repeats were ignored.

**Figure S6.** The increase in aligned reads as a function of the number of palindromes detected in libraries from ancient and historic samples.

Data were obtained from three previously published aDNA studies [6-8], see also main document. Only BWA alignments with a minimum MapQ value of 25 were considered. The increase in aligned reads was calculated by subtracting the number of alignments for libraries that included 3′-end terminal palindromes with more than three bases from those alignments for the same libraries that had these palindromes trimmed. Linear regression was used to calculate the R^2^ value.

**Table S1.** Clonal read abundance in Illumina TruSeq libraries from historic Atlantic cod samples.

The presence of clonal reads was assessed in reads *with* interrupted palindromes and those *without* using the MarkDuplicates command from Picard Tools v. 1.96 (http://picard.sourceforge.net/).

|  | Clonal reads (%) | |
| --- | --- | --- |
| Sample_ID | *With palindrome* | *Without palindrome* |
| 4-83 | 11.6 | 0.8 |
| 4-53 | 10.6 | 0.9 |
| 6-50 | 11.3 | 0.8 |
| 4-91 | 12.4 | 1.0 |
| W127 | 14.3 | 1.1 |
| W134 | 13.5 | 0.7 |
| 1586 | 10.6 | 0.7 |
| 676 | 30.1 | 0.8 |
| W131 | 3.4 | 0.8 |
| W135 | 4.7 | 0.8 |
| W137 | 5.2 | 1.8 |

**References**

1. Li H, Durbin R (2009) Fast and accurate short read alignment with Burrows-Wheeler transform. Bioinformatics 25: 1754-1760.

2. Ginolhac A, Rasmussen M, Gilbert MTP, Willerslev E, Orlando L (2011) mapDamage: testing for damage patterns in ancient DNA sequences. Bioinformatics 27: 2153-2155.

3. Jonsson H, Ginolhac A, Schubert M, Johnson PLF, Orlando L (2013) mapDamage2.0: fast approximate Bayesian estimates of ancient DNA damage parameters. Bioinformatics 29: 1682-1684.

4. Kibbe WA (2007) OligoCalc: an online oligonucleotide properties calculator. Nucleic Acids Research 35: W43-W46.

5. Benson G (1999) Tandem repeats finder: a program to analyze DNA sequences. Nucleic Acids Research 27: 573-580.

6. Rasmussen M, Li YR, Lindgreen S, Pedersen JS, Albrechtsen A, et al. (2010) Ancient human genome sequence of an extinct Palaeo-Eskimo. Nature 463: 757-762.

7. Rasmussen M, Guo X, Wang Y, Lohmueller K, Rasmussen S, et al. (2011) An Aboriginal Australian genome reveals separate human dispersals into Asia. Science 334: 94 - 98.

8. Seguin-Orlando A, Schubert M, Clary J, Stagegaard J, Alberdi MT, et al. (2013) Ligation Bias in Illumina Next-Generation DNA Libraries: Implications for Sequencing Ancient Genomes. Plos One 8: e78575.
